# Facile Synthesis, Sintering, and Optical Properties of Single-Nanometer-Scale SnO_2_ Particles with a Pyrrolidone Derivative for Photovoltaic Applications

**DOI:** 10.3390/ma17205095

**Published:** 2024-10-18

**Authors:** Wingki Mey Hendra, Naohide Nagaya, Yuto Hibi, Norimitsu Yoshida, Takashi Sugiura, Saeid Vafaei, Kazuhiro Manseki

**Affiliations:** 1 Graduate School of Natural Science and Technology, Gifu University, Yanagido 1-1, Gifu 501-1193, Japan; 2 Independent Researcher, Peoria, IL 61606, USA

**Keywords:** tin oxide (SnO_2_), nanoparticles, hydrolysis, TEM, solar cells

## Abstract

We investigate the preparation of mesoscopic SnO_2_ nanoparticulate films using a Sn(IV) hydrate salt combined with a liquid pyrrolidone derivative to form a homogeneous precursor mixture for functional SnO_2_ nanomaterials. We demonstrate that N-methyl-2-pyrrolidone (NMP) plays a crucial role in forming uniform SnO_2_ films by both stabilizing the hydrolysis products of Sn(IV) sources and acting as a base liquid during nanoparticle growth. The hydrolysis of Sn(IV) was controlled by adjusting the reaction temperature to as low as 110 °C for 48 h. High-resolution TEM analysis revealed that highly crystalline SnO_2_ nanoparticles, approximately 3–5 nm in size, were formed. The SnO_2_ nanoparticles were deposited onto F-doped SnO_2_ glass and converted into dense particle films through heat treatments at 400 °C and 500 °C. This pyrrolidone-based nanoparticle synthesis enabled the production of not only crystallized SnO_2_ but also transparent and uniform films, most importantly by controlling the slow hydrolysis of Sn(IV) and polycondensation only with those two chemicals. These findings offer valuable insights for developing stable and uniform electron transport layers of SnO_2_ in mesoscopic solar cells, such as perovskite solar cells.

## 1. Introduction

Nanosized tin oxide (SnO_2_) particles are widely recognized as functional materials with significant potential across diverse applications, including photovoltaics [1,2,3,4,5,6], gas sensors [7,8,9,10,11,12,13], batteries [14,15], and others [16,17]. The material’s characteristic wide band gap (typically 3.6 eV) and relatively high electron mobility (~200 cm^2^V^−1^s^−1^), along with its excellent transparency and chemical stability, make SnO_2_ particularly advantageous for various applications. The applications range from solar cells to X-ray detectors [18,19,20,21], where SnO_2_ and titanium dioxide (TiO_2_) are frequently used as electron transport materials.

This paper presents a novel method for synthesizing SnO_2_ nanomaterials and explores their potential applications in energy conversion, with a particular focus on photovoltaics. The global interest in perovskite solar cells (PSCs) has grown significantly in response to the increasing demand for next-generation renewable energy technologies. Recent advancements have driven the power conversion efficiency (PCE) of PSCs to exceed 26%, particularly in p-i-n structured devices [22]. Meanwhile, n-i-p structured PSCs, which employ SnO_2_ as the electron transport layer (ETL), have also attracted considerable attention [23]. Furthermore, recent innovations include the incorporation of molecular layers between the conductive oxide substrate and the perovskite absorber, aimed at enhancing device performance [24].

To fully exploit the cost advantages of PSCs, ongoing research must focus on the exploration of novel materials and the optimization of thin-film fabrication processes using a variety of precursor systems. One of the key advantages of PSCs is their compatibility with solution-based fabrication techniques, which enable the development of flexible devices on plastic substrates. However, high-temperature processing on glass substrates, particularly involving oxide nanoparticles, remains crucial for enhancing the performance and stability of PSCs. In this study, we present a facile synthesis method for SnO_2_ nanomaterials and explore their potential applications in pursuit of advancing PSC technology.

The functionalities of nanostructured metal oxides can be precisely controlled by adjusting the size and morphology of nanoparticles. A widely adopted approach to regulate nanoparticle growth in solution synthesis involves the use of structure-directing agents (SDAs), typically coordinating compounds that interact with metal ions within the nanoparticles [25]. One of the challenges remains in preventing nanoparticle agglomeration within metal oxide films. Addressing this issue requires the development of techniques that improve the quality of nanoparticulate thin films.

The synthesis of SnO_2_ nanoparticles involving tin–organic ligand complexes is a promising strategy. This approach necessitates meticulous regulation of the hydrolysis process of the tin precursor. Furthermore, in the fabrication of mesoscopic devices composed of interconnected nanoparticles, subsequent deposition and thermal treatment steps are critical for optimizing the film properties, as they significantly influence the stability of the resulting films.

When considering a manufacturing process in practical applications, factors such as the selection of reagents commonly used in the industry and the simplicity of the process play a crucial role in the development of a film formation process. We recently reported that the growth control and dispersion of SnO_2_ nanoparticles can be achieved at temperatures as low as 80 °C using L-phenylalanine methyl ester hydrochloride as an SDA, which is widely used in pharmaceutical and bio-related research [26]. The use of a hydrochloride ester offers the advantage of increasing the solubility of the SDA in an aqueous reaction medium due to the bulky phenyl group.

In this study, we investigate the synthesis of SnO_2_ nanoparticles using two simple chemical sources, Tin(IV) chloride pentahydrate and *N*-methyl-2-pyrrolidone (NMP), which undergo hydrolysis for approximately 48 h. We propose, for the first time, that such a slow hydrolysis in the nonaqueous reaction system facilitates crystallization even at low temperatures. Additionally, NMP is commonly employed in various industrial applications as a solvent, cleaning agent, and stripping agent. We evaluate the effectiveness of the pyrrolidone derivative in producing uniform SnO_2_ nanoparticle films through solution-based processes, including spin-coating and high-temperature sintering. The nanostructures of the resulting particles and thin films were characterized primarily using transmission electron microscopy (TEM). Furthermore, we demonstrate the application of these films as electron transport materials in perovskite solar cells. The TiO_2_-coated SnO_2_ layers prepared by sintering were also examined regarding oxygen vacancies in the newly synthesized SnO_2_ layers to elucidate their relationship with the performance of perovskite solar cells.

## 2. Materials and Methods

### 2.1. Chemicals

Tin (IV) chloride pentahydrate (SnCl_4_·5H_2_O, >98.0%) was acquired from FUJIFILM Wako Pure Chemical Corporation. *N*-Methyl-2-pyrrolidinone (99.5%) (NMP) was purchased from Sigma-Aldrich, Co., St. Louis, MO, USA. Ethanol (99.5%) was supplied from Kanto Chemical Co., Inc., Tokyo, Japan. H_2_O (resistivity: 18.2 MΩ·cm) was obtained using a Milli-Q^®^ integral water purification system (MERK Ltd., Tokyo, Japan). Concentrated nitric acid (60–61%) was procured FUJIFILM Wako Pure Chemical Corporation.

N,N-Dimethylformamide (DMF, dehydrated, >99.5%), dimethyl sulfoxide (DMSO, dehydrated, 99.0%), 2-propanol (IPA, dehydrated, >99.7%), titanium isopropoxide (TTIP, >97%), and acetonitrile (dehydrated, >99.5%) were obtained from Kanto Chemical Co., Inc. in Tokyo, Japan. Methylamine hydrobromide (MABr, >98.0%), cesium iodide (CsI, >99.0%), lead(II) iodide (PbI_2_, 99.99%), formamidine hydroiodide (FAI, 99.99%), lithium bis(trifluoromethanesulfonyl)imide (Li-TFSI, >98.0%), and formamidine hydrobromide (FABr, 99.99%) were sourced from Tokyo Chemical Industry Co., Ltd. in Tokyo, Japan. Spiro-MeOTAD, chlorobenzene (CB, anhydrous, 99.8%), and 4-tert-butylpyridine (TBP, 98%) were acquired from Sigma-Aldrich Co. in St. Louis, MO, USA.

All chemicals were used without further purification.

### 2.2. Synthesis of SnO_2_ Nanoparticles

A quantity of 2.31 g of tin(IV) chloride dihydrate, used as the tin source, was dissolved in NMP at a molar ratio of 1:5 in 3 mL. The solution became cloudy because of the addition of Sn source (SnCl_4_·5H_2_O). The mixture was then stirred at two different temperatures, 100 °C and 110 °C, for 48 h. The mixture gradually transformed into a yellowish, transparent solution.

**Figure 1:**
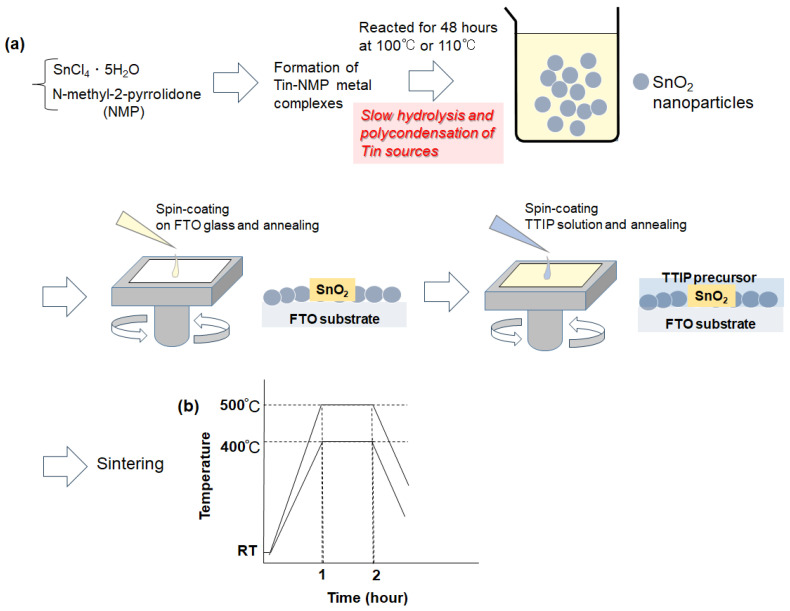
(**a**) Conceptual illustration of SnO_2_ thin-film formation via a pyrrolidone derivative-mediated synthesis of SnO_2_ nanoparticles through slow hydrolysis, followed by the sintering process of SnO_2_/TTIP substrates. (**b**) Sintering profiles of the SnO_2_/TTIP substrates.

We hypothesized that the high boiling point (202 °C) of NMP and the presence of its coordinating oxygen atom would enhance the dispersion of SnO_2_ nanoparticles in spin-coated films by facilitating the formation of multiple stable Sn(IV)–oxygen bonds. As illustrated in Figure 1a,b, the synthesis of SnO_2_ nanoparticles and their thin-film formation were carried out.

### 2.3. Preparation of SnO_2_-Based Thin Films

The nanofluid was the mixture of SnO_2_ nanoparticles and NMP as a base liquid. The spin-coating method is used to deposit the nanoparticles on a fluorine-doped tin oxide (FTO) substrate. In the first stage, nanoparticles deposited at 3000 rpm for 30 s and after that annealed for 5 min at 120 °C. After spin-coating onto an FTO glass substrate and heating at 120 °C, the sintering process was conducted at 400 °C and 500 °C to promote the fusion of nanoparticles within the films. Titanium tetraisopropoxide (TTIP), as a source of TiO_2_, was applied to the nanoparticle layer using the following procedure:

In the mixture of 13 mL of ethanol, 0.34 mL of distilled water, 3 drops of concentrated nitric acid, and 1 mL of TTIP solution was added dropwise. In the second stage, the TTIP solution was distributed on top of SnO_2_ layer using a two-step process. In step 1, deposited nanoparticles were coated at 1500 rpm, for 30 s, and in step 2, coating took place at 1000 rpm, for 60 s. Eventually, the coated substrate with SnO_2_/TTIP precursor was sintered in two different ways. The ramping rates from 20 °C were 6.33 °C/min and 8 °C/min in case 1 and 2, respectively. Maximum temperatures for case 1 and 2 were 400 °C and 500 °C, respectively. The period of sintering at maximum temperature was 1 h for both cases (see Figure 1b). An electric furnace, KDF 300 Plus (DENKEN-HIGHDENTAL, Kyoto, Japan), was used to conduct sintering.

### 2.4. Characterization of SnO_2_ Nanoparticles and Films

XRD measurement was carried out using the apparatus Rigaku RINT Ultima/PC with monochromated Cu–Kα radiation, Japan. The size of crystallite was determined using Scherrer Equation (1), where D, K, λ, and θ present the crystallite size, Scherrer constant (0.90), X-ray wavelength (1.54 Å), and Bragg angle, respectively.

D = Kλ/βcosθ(1)

SnO_2_ particles were characterized using SEM-EDX (S-4800, Hitachi High-Tech Corporation, Tokyo, Japan) and TEM (JEM-2100, JEOL, Tokyo, Japan). A confocal Raman microscope, RAMANtouch (Nanophoton Corp., Tokyo, Japan), was used for the characterization. Laser light of 532 nm was used to irradiate the powder samples, and the laser power was 10 mW/cm^2^. Diffuse reflectance spectra were also measured using a UV-Vis Spectrophotometer (V-700, Jasco, Tokyo, Japan). The SnO_2_-based films were analyzed by XPS (XPS; ULVAC, Quantera SXM, Kanagawa, Japan). IR measurements of solution samples were also carried out using JASCO FT-IR-4700, Japan.

### 2.5. Fabrication and Evaluation of Perovskite Solar Cells

The perovskite layer, Cs_0.05_MA_0.1_FA_0.85_PbI_2.9_Br_0.1_·0.05PbI_2_, was deposited onto the metal oxide substrate via a modified procedure previously detailed in our earlier study [27], conducted inside a nitrogen-filled glove box with a relative humidity of less than 10%. Prior to the deposition of the perovskite solution, the FTO-coated substrate was pre-heated to 80 °C using a hot plate. The perovskite precursor solution was prepared as outlined in our earlier work [26], where 0.14 mmol (16 mg) of MABr, 1.45 mmol (669 mg) of PbI_2_, 1.19 mmol (205 mg) of FAI, and 0.07 mmol (18 mg) of CsI were dissolved in a solvent mixture of 80 vol% DMF (0.8 mL) and 20 vol% DMSO (0.2 mL). The solution was stirred at 80 °C for 2 h. Following this, the substrate was placed into a spin-coater, and 100 μL of the perovskite solution was dispensed onto it. A two-step spin-coating process was carried out at 1000 rpm for 10 s, followed by 6000 rpm for 30 s. After 25 s of spin-coating, 200 μL of CB was introduced. The substrate was then heated at 100 °C for 1 h on a hot plate to promote perovskite layer formation and subsequently allowed to cool to room temperature naturally.

Simultaneously, a solution of FABr (30 mM) was prepared by dissolving 0.12 mmol (0.0148 g) of FABr in 4 mL of IPA. After the formation of the perovskite layer, 100 μL of this FABr solution was spin-coated onto the layer at 3000 rpm for 30 s. The substrate was then heated at 80 °C for 10 min on a hot plate, followed by cooling to room temperature. For the deposition of the hole-transport material, Spiro-OMeTAD, 0.037 mmol (0.045 g) was dissolved in 0.5 mL of CB. Additionally, 0.18 mmol (0.052 g) of Li-TFSI was dissolved in 0.1 mL of acetonitrile, and 10 μL of this acetonitrile solution, along with 17.75 μL of TBP, was added to the Spiro-OMeTAD solution. A 60 μL aliquot of this prepared solution was then spin-coated onto the perovskite layer at 4000 rpm for 20 s. The substrate was left in ambient air for 24 h. Finally, a gold layer was deposited onto the Spiro-OMeTAD using a thermal evaporation technique, following the method previously established by our group as referenced [28].

The current–voltage (I-V) curves were obtained under incident light with an intensity of 100 mW/cm^2^, simulating full sunlight using a solar simulator (YSS-80A, Yamashita Denso, Tokyo, Japan) in combination with a potentiostat (HSV-110, Meiden Hokuto, Tokyo, Japan) for analysis. The active area of the device was regulated at 0.09 cm^2^. The I-V curves were measured at a scan rate of 50 mV/s. A cross-sectional image of the device was obtained using scanning electron microscopy (SEM) (S-4800, Hitachi High-Tech Corporation, Tokyo, Japan).

## 3. Results and Discussions

**Figure 2:**
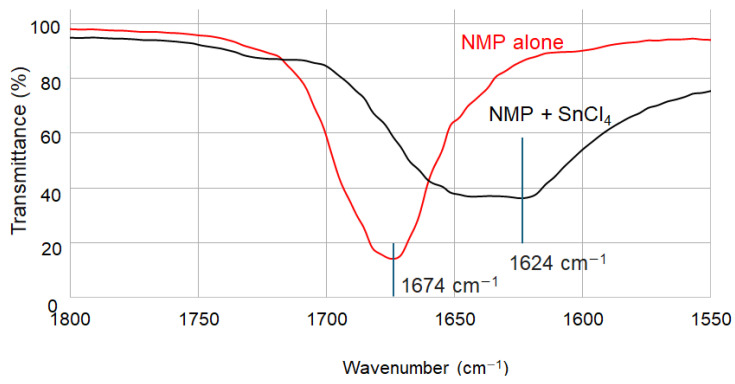
IR spectra of NMP alone and the mixture of NMP and SnCl_4_.

To investigate the metal complexation between Sn(IV) and NMP, we conducted infrared (IR) spectroscopy measurements on solution samples containing either NMP alone or the Sn(IV)-NMP mixture (without heating). As shown in Figure 2, the observed shift in the C = O stretching frequency in the Sn(IV)-NMP solution provides evidence for the formation of a Sn(IV)-O bond in solution [29].

**Figure 3:**
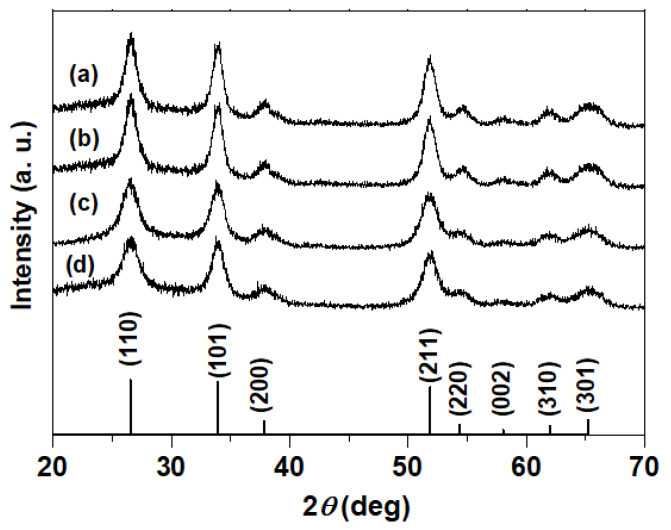
XRD pattern of SnO_2_ samples showing the effects of reaction and sintering temperatures on the SnO_2_ samples in (**a**–**d**). Reaction temperatures are 100 °C and 110 °C, while sintering temperatures are 400 °C and 500 °C: (**a**) reaction at 110 °C/sintering at 500 °C, (**b**) reaction at 100 °C/sintering at 500 °C, (**c**) reaction at 110 °C/sintering at 400 °C, and (**d**) reaction at 100 °C/sintering at 400 °C. The database (JCPDS: 00-041-1445) of Cassiterite SnO_2_ is also presented at the bottom.

To characterize the formation of SnO_2_ during solution synthesis, X-ray diffraction (XRD), Raman spectroscopy, and transmission electron microscopy (TEM) were conducted. Figure 3 shows the XRD patterns of the sintered SnO_2_ samples. In panels (a–d) of Figure 3, the reaction temperatures for the SnO_2_ solution were set at 100 °C and 110 °C, while the sintering temperatures were 400 °C and 500 °C. All observed diffraction peaks correspond to rutile-type SnO_2_ (JCPDS: 00-041-1445), with no significant peak shifts observed due to variations in reaction or sintering temperatures. A slight increase in peak intensity was noted at the higher sintering temperature of 500 °C, indicating improved crystallinity. The crystallite sizes of each sample were calculated using Scherrer’s equation, as detailed in Table 1. When deposited nanoparticles were sintered at 500 °C, both reaction temperatures (100 °C and 110 °C) led to an increase in crystallite size of 1–2 nm across all crystal planes compared to samples sintered at 400 °C. This increase in crystallite size is likely due to more pronounced necking between particles at higher sintering temperatures. Obviously, higher crystal growth is expected at higher maximum temperature. As a result, the recombination of electrons and holes at the grain boundaries of nanoparticles is expected to be suppressed, leading to improved light-to-electricity conversion efficiency.

Although the crystallite sizes increased as the sintering temperature was raised from 400 °C to 500 °C, the absence of significant peak shifts indicates that there are no substantial changes in the lattice structures for the samples synthesized between 100 °C and 110 °C, or sintered between 400 °C and 500 °C.

**Figure 4:**
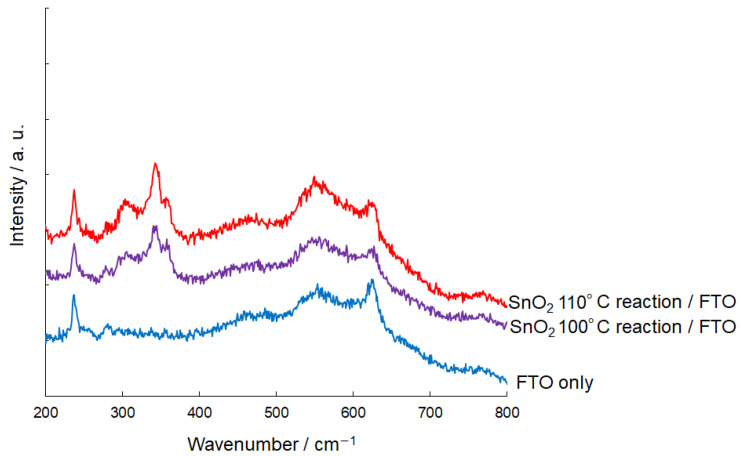
Raman spectra of as-deposited SnO_2_ films annealed on FTO substrates are presented. The SnO_2_ films were synthesized through reactions conducted at 100 °C and 110 °C. For comparison, the Raman spectrum of the FTO substrate is also shown.

The Raman spectra of as-synthesized nanoparticles in thin films were investigated, as shown in Figure 4. For comparison, the Raman spectra of the FTO substrate surface were also measured. For nanoparticles synthesized at 100 °C and 110 °C, several peaks were detected between 300 and 400 cm^−1^, which are not typically observed in bulk single-crystal or polycrystalline samples [30]. The characteristic vibration modes of E_g_ and A_1g_ for the obtained SnO_2_ nanoparticles could not be distinctly assigned due to the overlap with the Raman modes of the SnO_2_ of FTO substrate. Cheng et al. also reported Raman scattering near 330 cm^−1^ and highlighted that these peaks are sensitive to the crystal surface area, influenced by the small crystal sizes [30]. The grain sizes and local disorder may significantly affect the Raman scattering, potentially leading to the emergence of new Raman modes in nano-crystalline samples.

**Figure 5:**
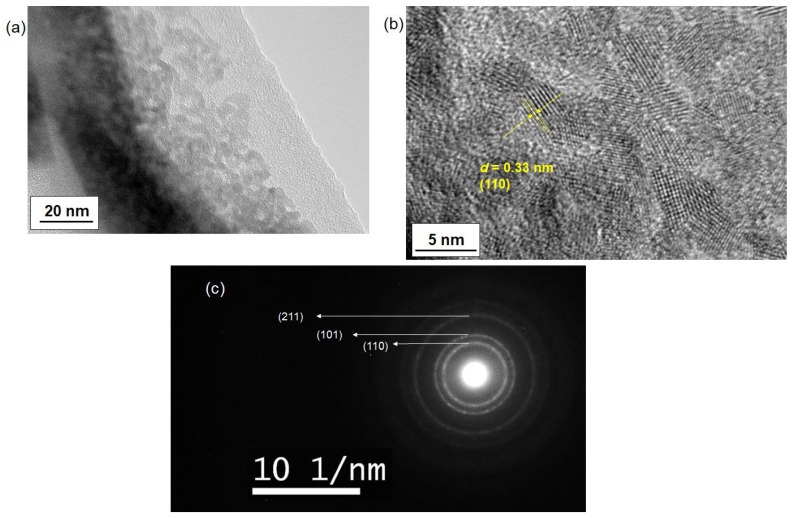
TEM images of as-synthesized SnO_2_ nanoparticles at different magnifications (**a**,**b**). The nanoparticles were synthesized through a reaction at 100 °C using tin chloride pentahydrate. In (**b**), the d-spacing of the lattice fringes corresponding to the (110) plane of SnO_2_ is displayed. Image (**c**) presents the selected area diffraction (SAD) pattern of the same SnO_2_ sample.

A transmission electron microscope (TEM) was used to confirm the crystallization of SnO_2_ nanoparticles. TEM images of the as-synthesized nanoparticles reacted at 100 °C are shown in Figure 5a,b. Figure 5a reveals the uniform growth of the nanoparticles. Lattice fringes, as shown in Figure 5b, were observed, while Figure 5c presents a selected area diffraction (SAD) pattern, confirming the formation of SnO_2_ nanoparticles. The nanoparticles were approximately 3–5 nm in size, which agrees with the crystallite sizes obtained from the XRD data. Additionally, the observation of a single set of lattice fringes in (b) corresponding to a specific crystal plane across the entire particle indicates the single-crystal nature of the SnO_2_ nanoparticles. The SAD pattern displayed characteristic rings corresponding to the (110), (101), and (211) planes, indicating the presence of SnO_2_. The observation of some diffraction spots also indicated the highly crystallized nature.

An excess amount of NMP relative to the metal ion sources likely suppressed the growth of larger nanoparticles through the formation of metal–ligand coordination bonds, resulting in the synthesis of single-nanometer-scale SnO_2_ particles as small as 5 nm.

**Figure 6:**
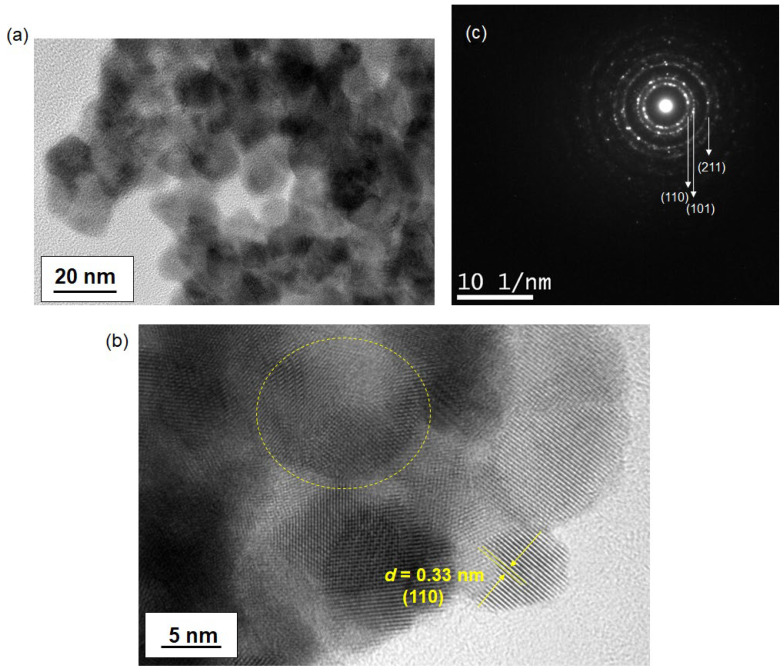
TEM images of SnO_2_ nanoparticles sintered at 500 °C. Images (**a**,**b**) show the heat-treated nanoparticles at different magnifications. The nanoparticles were synthesized through a 100 °C reaction. In (**b**), the d-spacing of the lattice fringes of SnO_2_ is depicted, with the dotted circle highlighting the formation of polycrystalline nanoparticles. Image (**c**) displays the selected area diffraction (SAD) pattern of the same SnO_2_ sample.

The structure of the sintered sample at 500 °C was also investigated, as shown in Figure 6. Unlike the as-synthesized nanoparticles, the sintered sample exhibited densely packed nanoparticles due to the heating process, which enhanced the connectivity between particles, as depicted in Figure 6b. The SAD pattern in Figure 6c confirmed the presence of SnO_2_ with diffraction rings corresponding to the (110), (101), and (211) planes. The polycrystalline nature of the sample was evident, as shown by the lattice fringes in Figure 6b, highlighted with a dotted yellow line. Overall, the sintered sample displayed relatively larger particles compared to the non-sintered sample, likely due to nanoparticle growth during the high-temperature treatment at 500 °C.

**Figure 7:**
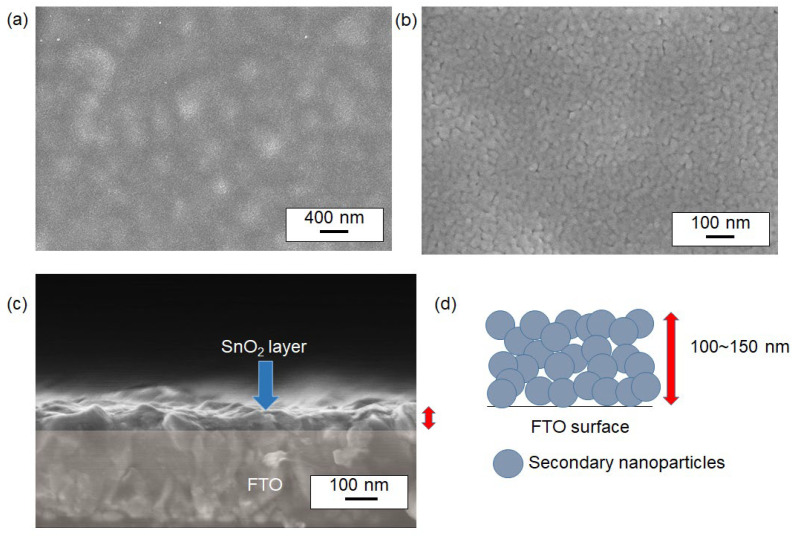
SEM images of the surface of SnO_2_ thin films sintered at 400 °C. Images (**a**,**b**) show the nanoparticles at two different magnifications. The SnO_2_ nanoparticles were synthesized via a 100 °C reaction. Image (**c**) presents a cross-sectional view of the film. A conceptual illustration of the nanoparticle stack from (**c**) is shown in (**d**). The red arrows in (**c**,**d**) indicate the thickness of the film of the same interest.

**Figure 8:**
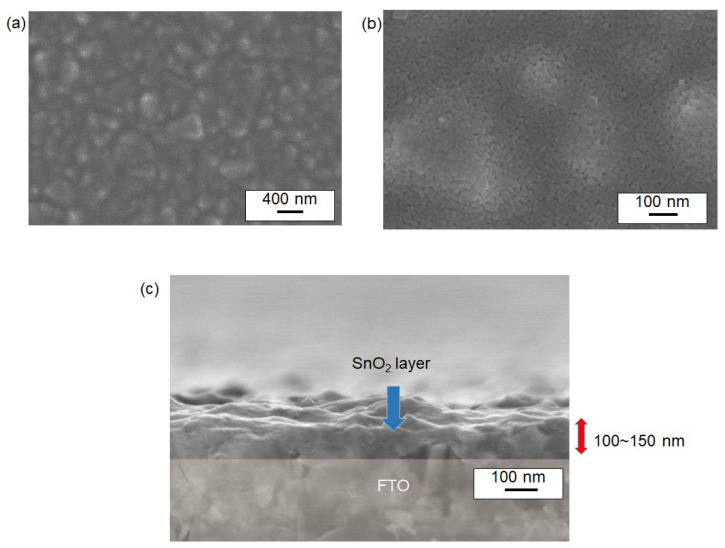
SEM images of the surface of SnO_2_ thin films sintered at 500 °C. Images (**a**,**b**) display the nanoparticles at different magnifications. The SnO_2_ nanoparticles were synthesized from a reaction at 100 °C. Image (**c**) provides a cross-sectional view of the film. The red arrows in (**c**) highlight the thickness of the film.

The surface cross-sectional structure of sintered SnO_2_ nanoparticle films was visualized in Figure 7 and Figure 8, showing SEM data of films synthesized at two different sintering temperatures: 400 °C and 500 °C (without a titanium source). Both conditions revealed similar porous nanostructures. For instance, Figure 7 shows a sample sintered at 400 °C, where the textured FTO surface was fully covered with SnO_2_ nanoparticles. Notably, nanostructured domains consisting of particulates with dimensions of approximately 20–30 nm formed porous films, as seen in Figure 7a,b. Based on the previously discussed TEM images, these larger domains are suggested to be assemblies of smaller primary particles (secondary nanoparticles) approximately 3–5 nm in size. The cross-sectional SEM image in Figure 6c indicates that the deposited film has an approximate thickness of 100–150 nm, suggesting that an average of 5–6 layers of nanoparticles were stacked on the FTO surface, as illustrated in Figure 7d. Similar nanostructures were observed for films sintered at 500 °C as shown in Figure 8.

**Figure 9:**
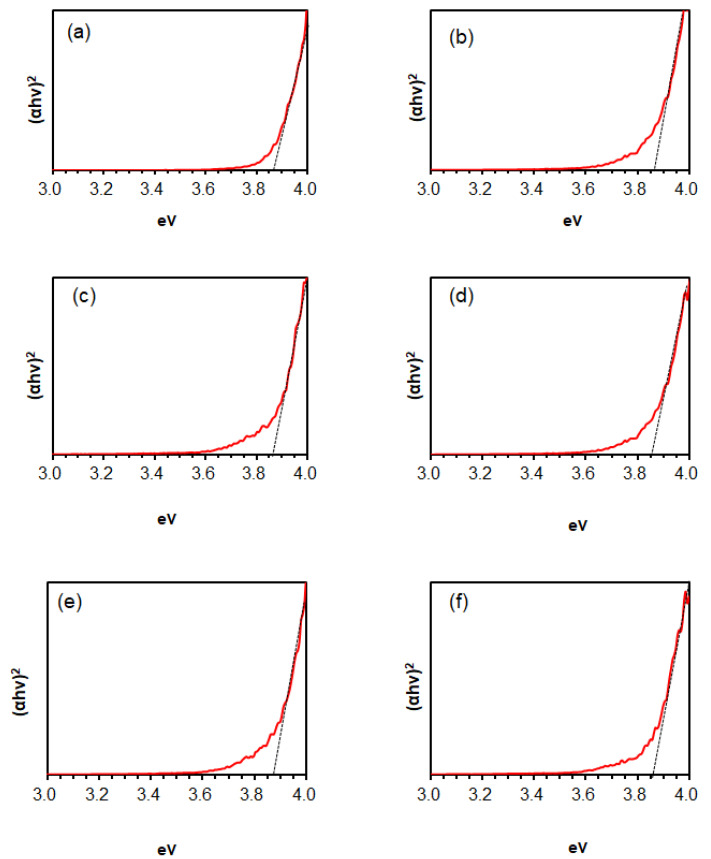

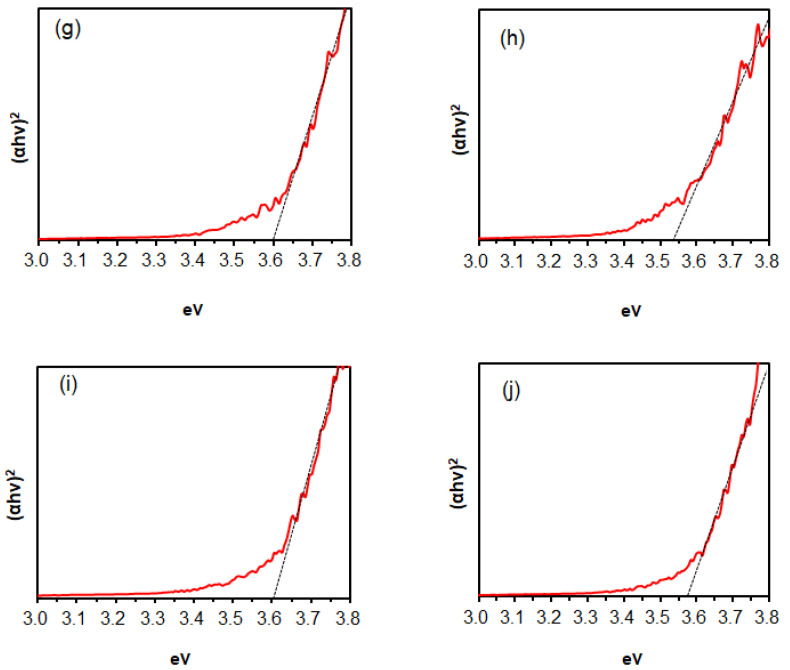
Diffuse reflectance spectra of SnO_2_ films. Panels (**a**–**j**) display the effects of reaction and sintering temperatures with and without TTIP on the SnO_2_ samples. Reaction temperatures are 100 °C and 110 °C, and sintering temperatures are 400 °C and 500 °C: (**a**) reaction at 100 °C, (**b**) reaction at 110 °C, (**c**) reaction at 100 °C/sintering at 400 °C without TTIP, (**d**) reaction at 100 °C/sintering at 500 °C without TTIP, (**e**) reaction at 110 °C/sintering at 400 °C without TTIP, (**f**) reaction at 110 °C/sintering at 500 °C without TTIP, (**g**) reaction at 100 °C/sintering at 400 °C with TTIP, (**h**) reaction at 100 °C/sintering at 500 °C with TTIP, (**i**) reaction at 110 °C/sintering at 400 °C with TTIP, and (**j**) reaction at 110 °C/sintering at 500 °C with TTIP.

From the diffuse reflectance spectra shown in Figure 9, the optical band gap (*E_g_*) of the SnO_2_ thin film samples was estimated by Tauc Equation (2), where *h*, *ν*, *α*, and *A* represent the Planck constant, photon frequency, absorption coefficient, and constant, respectively.

(*αhν*)^2^ = *A*(*hν* − *E_g_*) (2)

As shown in Table 2, *E_g_* values were determined to be 3.85–3.87 eV (a–f), regardless of the reaction temperature of the SnO_2_ solution, or the sintering temperature. These relatively large values are consistent with previously reported data and are attributed to the quantum confinement effect observed in single-nanometer-scale crystals [31]. In a previous study, Matysiak et al. also highlighted that changes in the band gaps of SnO_2_ nanoparticles are influenced by the synthesis method, including sol–gel, spray pyrolysis, hydrothermal, thermal evaporation, and electrospinning techniques. They demonstrated that nanoparticles produced via electrospinning exhibited an increased band gap (~3.9 eV) due to a pronounced quantum confinement effect. In contrast, such increases in band gap have been predominantly reported for high-temperature synthesis methods, such as hydrothermal processes and calcination. Notably, our study shows that the slow hydrolysis process employed for the synthesis of SnO_2_ nanoparticles induces quantum confinement effects even at relatively low temperatures, as low as 100 °C.

Regarding the application of SnO_2_ layers in perovskite solar cells (PSCs), we found that the direct integration of the perovskite precursor solution onto the SnO_2_ layer was unsuccessful due to low affinity during the spin-coating process. As an alternative approach to achieve favorable electron transport layer (ETL)/perovskite interfaces, we coated the SnO_2_ layer with a titanium isopropoxide (TTIP) solution as a source of TiO_2_, resulting in the formation of an ETL/perovskite layer.

**Figure 10:**
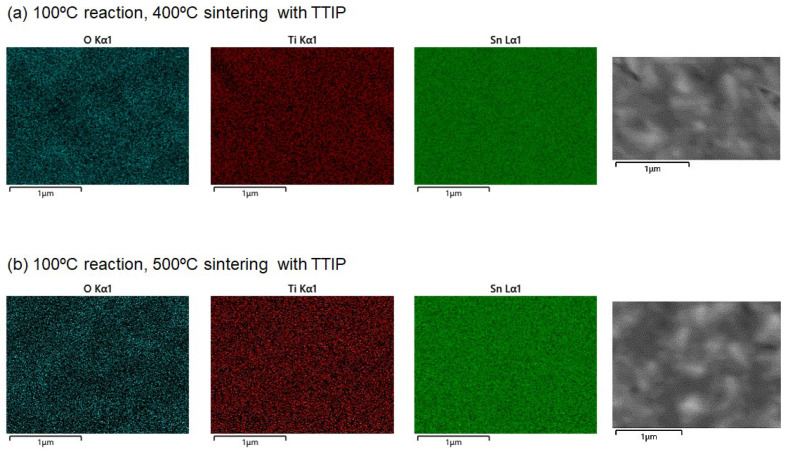

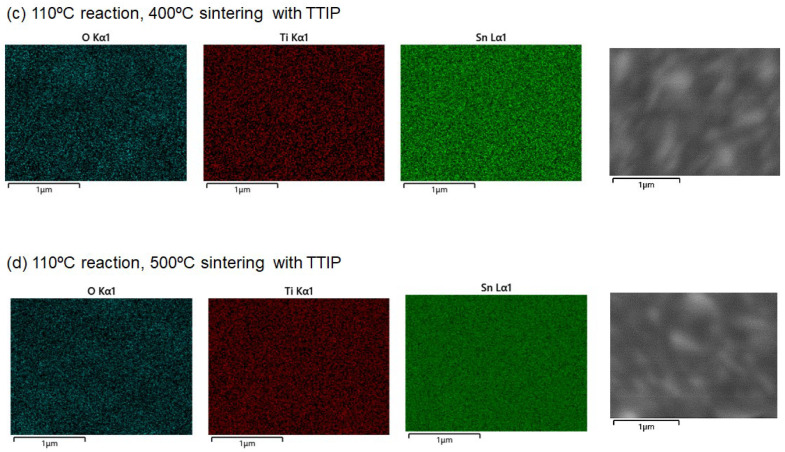
(**a**–**d**) present elemental maps obtained from energy-dispersive X-ray spectroscopy (EDS), showing the distribution of O, Ti, and Sn in the SnO_2_-based films. The corresponding SEM images for the EDS measurements are also shown.

The effect of an additional TTIP layer on the SnO_2_ underlayer during the sintering process was also examined. Notably, the band gap narrowed to 3.53–3.60 eV at both reaction temperatures of 100 °C and 110 °C. The band gap of TiO_2_ is known to range between 3.0 and 3.2 eV, which is smaller than that of SnO_2_. The formation of a SnO_2_/TiO_2_ solid solution may be responsible for the reduced band gap in the double-layered substrates [32]. We obtained the EDX elemental mappings for the four samples, as shown in Figure 10. The analysis clearly indicates a uniform distribution of Ti ions, suggesting the formation of SnO_2_/TiO_2_ solid solution.

**Figure 11:**
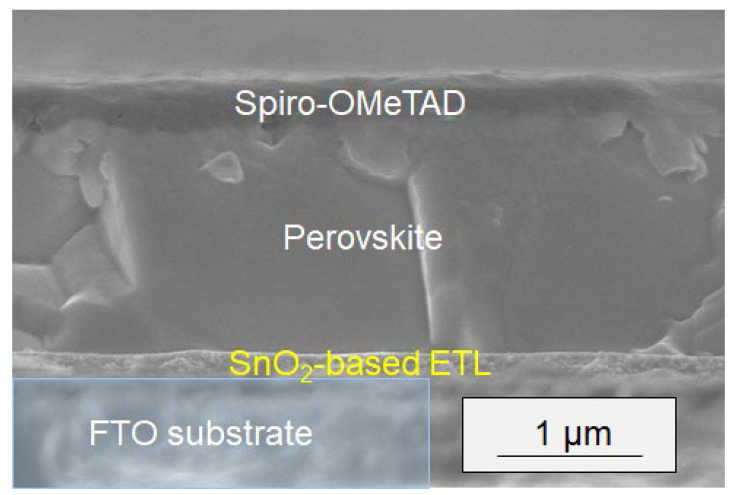
Cross-sectional SEM image of a PSC incorporating a newly prepared electron transport layer (SnO_2_-based ETL). The ETL was fabricated through a 100 °C reaction of SnO_2_ followed by sintering at 500 °C. Highlighted in yellow is the area of SnO_2_-based ETL deposited on the FTO substrate.

The sintered SnO_2_ and double-layered substrates were used for the deposition of a halide perovskite layer to evaluate the performance of PSCs with SnO_2_ as the electron transport layer (ETL). Due to the low affinity of the pure SnO_2_ surface in our samples, perovskite deposition by the spin-coating process was unsuccessful. In contrast, the double-layered substrates successfully formed a perovskite layer on the FTO substrate, which was then utilized to fabricate devices for further evaluation. In principle, the perovskite compound captures incident photons, while the ETL functions as an electron-extracting layer. Figure 11 shows a cross-sectional SEM image of a representative device, clearly displaying a mesoporous layer of nanoparticles beneath the bulk perovskite layer.

**Figure 12:**
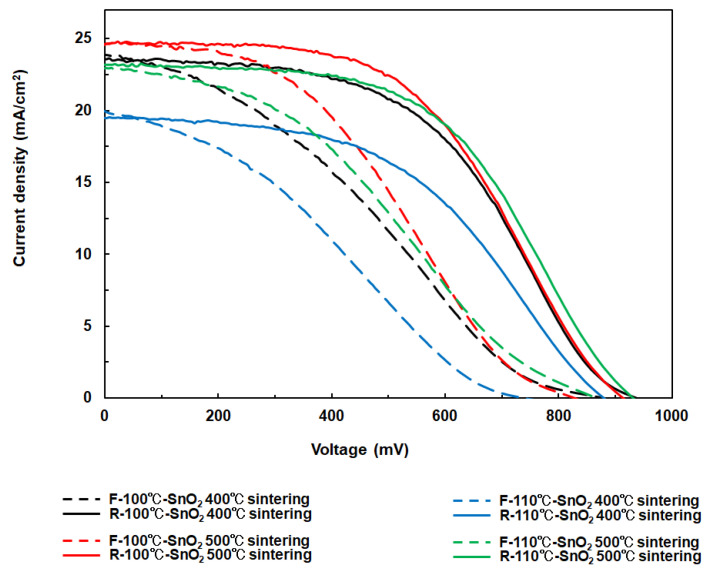
I-V curves of PSCs with different ETLs. The measurements were carried out under simulated sunlight (100 mW/cm^2^).

Current–voltage (I–V) curves were measured under simulated one sun illumination, assessing short-circuit current density (J_sc_), open-circuit voltage (V_oc_), fill factor (FF), and light-to-electricity conversion efficiency (η) (Figure 12 and Table 3). The η value was calculated using Equation (3), where iph and I represent the integral photocurrent density and the intensity of the illuminated light (I = 100 mW/cm^2^), respectively. Preliminary evaluation indicated that sintering at 500 °C resulted in a higher η compared to sintering at 400 °C for both reaction temperatures of 100 °C and 110 °C, likely due to the improved crystallinity of the ETLs. The relatively low efficiency (η: ~11.57%) is likely attributable to imperfect optimization of the perovskite layer, as evidenced by the smaller grain size of the perovskite crystals relative to the layer thickness (Figure 11). It is well known that these grain boundaries can induce significant recombination of electrons and holes, thereby reducing the overall efficiency. Further enhancement of η is anticipated through full optimization of the growth conditions of the perovskite layer, which should be adjusted based on the ETL layer to form the optimal ETL/perovskite interface.

η = (iph × V_oc_ × FF)/I (3)

**Figure 13:**
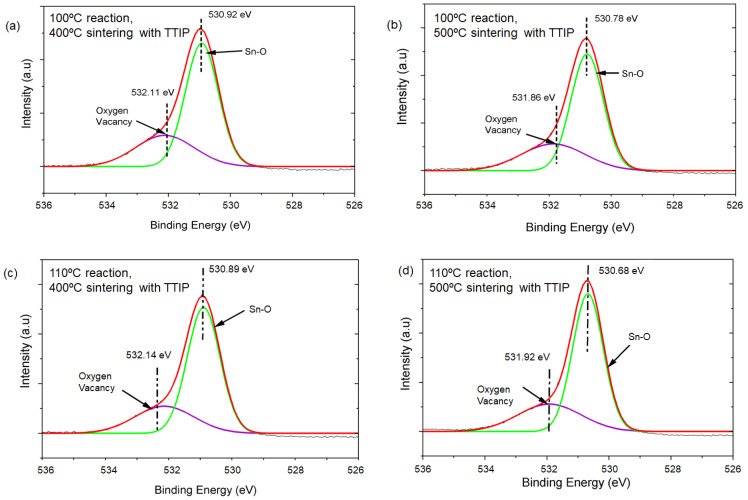
(**a**–**d**) present the XPS spectra of the O 1s for the SnO_2_ films sintered with TTIP.

To provide more insight into the relationship between the synthesized SnO_2_ nanomaterials and solar cell performance, we conducted XPS measurements on all TiO_2_-coated SnO_2_ substrates (Figure 13 and Table 4). Our findings indicate that the oxygen vacancy levels are comparable or slightly higher for the samples sintered at 500 °C. Based on these results, we believe that the improved J_sc_ and FF values for the 500 °C samples are likely due to enhanced nanoparticle interconnection during the higher-temperature process, which promotes increased electron transport, as confirmed by XRD and TEM analyses.

## 4. Conclusions

We discussed a facile synthesis of highly crystallized SnO_2_ nanoparticles with dimensions of approximately 3–5 nm in solution, achieved using two chemicals—Sn(IV) pentahydrate and *N*-methyl-2-pyrrolidone (NMP). This approach enabled the formation of a homogeneous solution containing SnO_2_ nanoparticles at temperatures as low as 110 °C. Notably, NMP played a dual role in the process: it stabilized the Sn(IV) source through complexation and served as a base solvent, facilitating the growth of SnO_2_. The hydrolysis of the Sn(IV) sources was effectively controlled by adjusting the temperature and the concentrations of the starting materials. A transparent and uniform SnO_2_ film was obtained by controlling the slow hydrolysis and polycondensation for 48 h. Sintering of the SnO_2_ nanoparticles at maximum temperatures of 400 °C and 500 °C was performed to form a dense particle film on a conductive glass substrate. Cross-sectional SEM imaging after spin-coating and sintering revealed the formation of a thin film, 100–150 nm in thickness, composed of assembled nanoparticles approximately 20–30 nm in size.

Remarkably, the band gaps of the as-synthesized SnO_2_ films were approximately 3.85–3.87 eV due to the quantum confinement effect and were significantly reduced by approximately 0.3 eV, particularly after sintering with an additional layer of TTIP. When applied as an electron transport layer in perovskite solar cells, the SnO_2_ nanoparticle film sintered at 500 °C demonstrated improved light-to-electricity conversion efficiency, highlighting the critical importance of sintering strategies for small SnO_2_ nanoparticles. Our NMP-based solution approach, which emphasizes the crystal growth of SnO_2_ nanoparticles through slow hydrolysis and sintering, offers valuable insights for the development of stable and uniform SnO_2_ electron transport layers in various mesoscopic-type solar cells.

## Figures and Tables

**Table 1:** Crystallite sizes of SnO_2_ samples estimated from XRD analysis. The data correspond to the samples depicted in Figure 3a–d.

	SnO_2_ Samples	Crystallite Sizes for Each Plane (nm)		
		(110)	(101)	(211)
(a)	110 °C reaction/500 °C Sintering	5.8	6.7	6.3
(b)	100 °C reaction/500 °C Sintering	5.7	6.9	6.7
(c)	110 °C reaction/400 °C Sintering	4.3	5.3	4.8
(d)	100 °C reaction/400 °C Sintering	4.0	5.5	4.9

**Table 2:** Band gaps of SnO_2_ films from the samples shown in Figure 8, estimated from diffuse reflectance spectra.

SnO_2_ Films (Nanoparticles Synthesis/Heat Treatment)	Bandgap (eV)
100 °C reaction, Annealing at 120 °C only	3.86
110 °C reaction, Annealing at 120 °C only	3.85
100 °C reaction, Sintering at 400 °C without TTIP	3.86
100 °C reaction, Sintering at 500 °C without TTIP	3.85
110 °C reaction, Sintering at 400 °C without TTIP	3.87
110 °C reaction, Sintering at 500 °C without TTIP	3.86
100 °C reaction, Sintering at 400 °C with TTIP	3.60
100 °C reaction, Sintering at 500 °C with TTIP	3.53
110 °C reaction, Sintering at 400 °C with TTIP	3.60
110 °C reaction, Sintering at 500 °C with TTIP	3.57

**Table 3:** I-V parameters of PSCs utilizing SnO_2_-based ETLs. All ETLs were prepared using TTIP. ‘R’ and ‘F’ denote reverse and forward scanning conditions, respectively, during the I-V curve measurements.

SnO_2_ films	V_oc_ (mV)	J_sc_ (mA/cm^2^)	FF	H (%)
R-100 °C-SnO_2_ 400 °C sintering	939	23.58	0.491	10.88
F-100 °C-SnO_2_ 400 °C sintering	882	23.86	0.301	6.33
R-100 °CSnO_2_ 500 °C sintering	915	24.64	0.513	11.57
F-100 °CSnO_2_ 500 °C sintering	829	24.62	0.384	7.84
R-110 °CSnO_2_ 400 °C sintering	881	19.47	0.488	8.37
F-110 °CSnO_2_ 400 °C sintering	742	19.97	0.310	4.60
R-110 °CSnO_2_ 500 °C sintering	932	23.16	0.529	11.42
F-110 °CSnO_2_ 500 °C sintering	866	22.92	0.350	6.95

**Table 4:** (a–d) Contents of oxygen species estimated from O 1s XPS spectra.

	Samples	Oxygen Vacancy (%)	Sn-O (%)
(a)	100 °C reaction, 400 °C sintering with TTIP	31.1	68.9
(b)	100 °C reaction, 500 °C sintering with TTIP	31.3	68.7
(c)	110 °C reaction, 400 °C sintering with TTIP	28.0	72.0
(d)	110 °C reaction, 500 °C sintering with TTIP	29.3	70.7

## Data Availability

The original contributions presented in the study are included in the article, further inquiries can be directed to the corresponding author.
